# Supplementary material for: Determination of Methyl Methanesulfonate and Ethyl Methylsulfonate in New Drug for the Treatment of Fatty Liver Using Derivatization Followed by High-Performance Liquid Chromatography with Ultraviolet Detection
Source: Molecules. 2022 Mar 17;27(6):1950. doi: 10.3390/molecules27061950 (PMC8951586; doi:10.3390/molecules27061950)
Supplement: Supplementary file 1 [file molecules-27-01950-s001.zip › molecules-1620621-supplementary.pdf]

Supporting information

Determination of Methyl Methanesulfonate and Ethyl Methylsulfonate in New Drug for the Treatment of Fatty Liver Using Derivatization Followed by High–Performance Liquid Chromatography with Ultraviolet Detection

Table of Contents

Figure S1. HPLC chromatograms of different derivatization solvents……………….........S1

Figure S2. HPLC chromatograms of temperatures and times of derivatization reaction...S2

Figure S3. HPLC chromatograms of concentrations of derivatization reagent added……S3

Figure S4. HPLC chromatograms of concentrations of NaOH added………….………S4

Figure S5. HPLC chromatograms of concentrations of Na_2CO_3 added…...…….…………S5

Figure S6. HPLC chromatograms of amount of Et_3N added…..……………………….…...S6

Figure S7. HPLC chromatograms of types of base……………………..……….……….....S6

Figure S8. HPLC chromatograms of samples……….…………….…………….……….....S7

Figure S9. GC-MS chromatograms of samples……………………………………………..S7

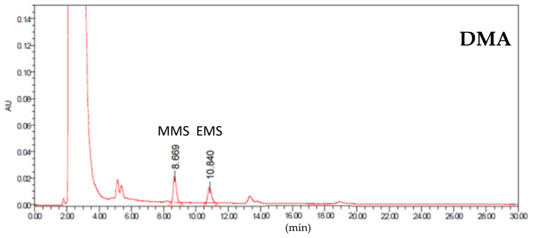

Retention time (min)	Peak area (μV*s)	Peak height (μV)
8.669	257607	19592
10.840	184369	11789

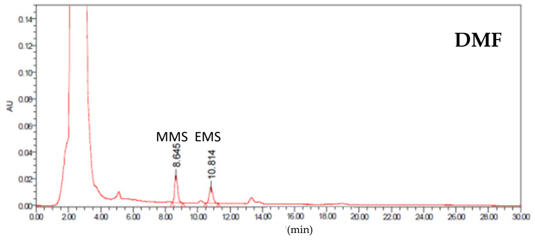

Retention time (min)	Peak area (μV*s)	Peak height (μV)
8.645	263365	20731
10.814	188895	12234

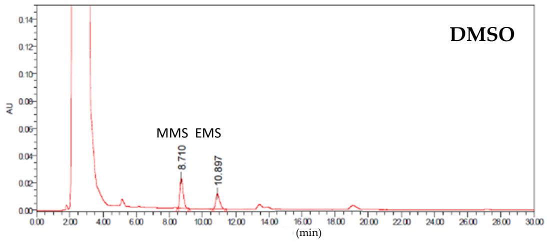

Retention time (min)	Peak area (μV*s)	Peak height (μV)
8.710	298417	22374
10.897	180206	11261

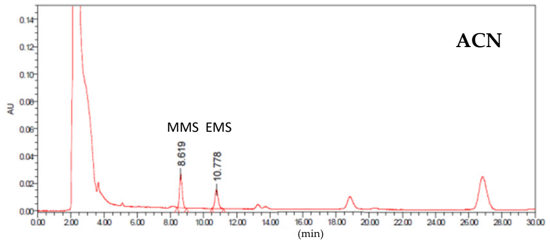

Retention time (min)	Peak area (μV*s)	Peak height (μV)
8.619	269541	24963
10.778	188166	13969

Figure S1. HPLC chromatograms of different derivatization solvents.

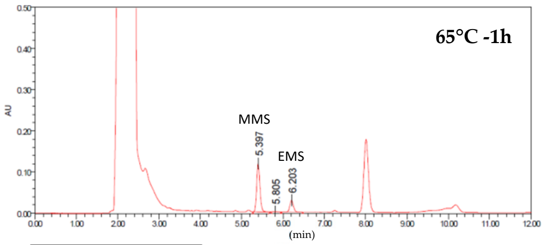

Retention time (min)	Peak area (μV*s)
5.397	646482
6.203	177266

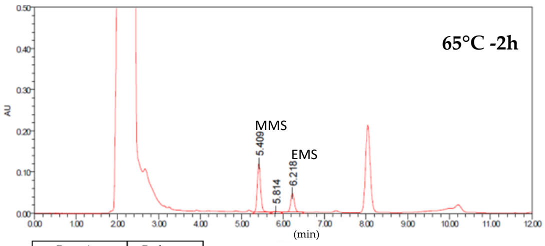

Retention time (min)	Peak area (μV*s)
5.409	646668
6.218	283482

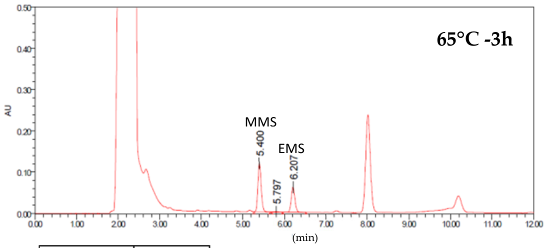

Retention time (min)	Peak area (μV*s)
5.400	650787
6.207	376283

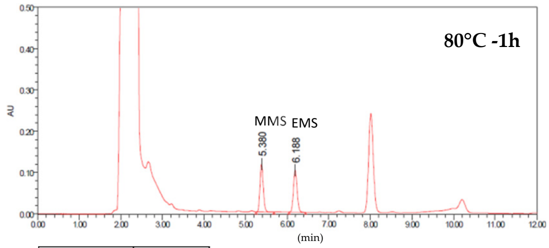

Retention time (min)	Peak area (μV*s)
5.380	640306
6.188	627868

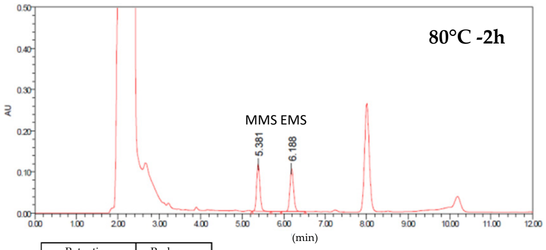

Retention time (min)	Peak area (μV*s)
5.381	630625
6.188	644338

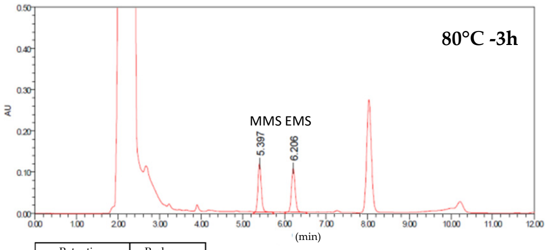

Retention time (min)	Peak area (μV*s)
5.397	643394
6.206	650608

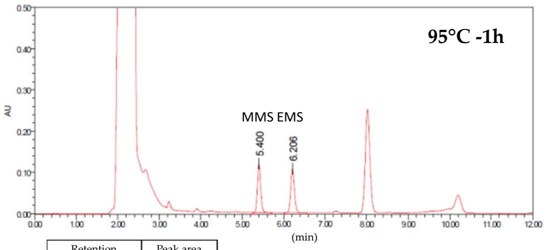

Retention time (min)	Peak area (μV*s)
5.400	645691
6.206	653189

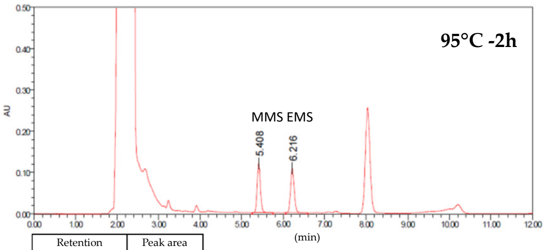

Retention time (min)	Peak area (μV*s)
5.408	654049
6.216	658211

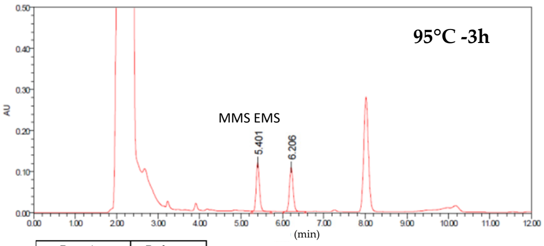

Retention time (min)	Peak area (μV*s)
5.401	648320
6.206	658690

Figure S2. HPLC chromatograms of temperatures and times of derivatization reaction.

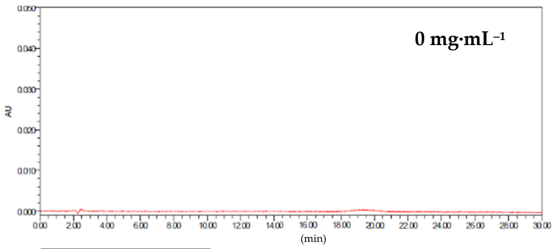

Retention time (min)	Peak area (μV*s)
-	-
-	-

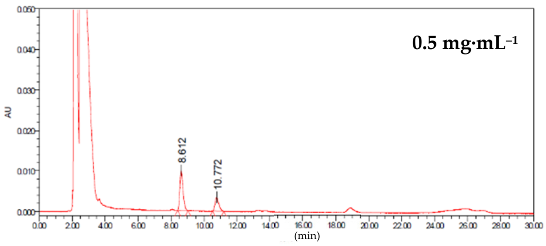

Retention time (min)	Peak area (μV*s)
8.612	130346
10.772	55169

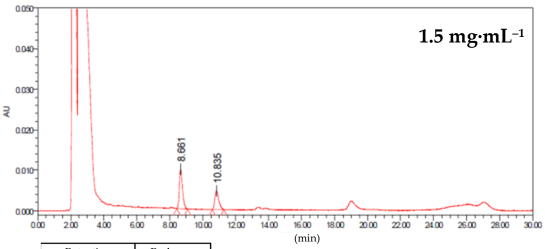

Retention time (min)	Peak area (μV*s)
8.661	130056
10.835	75544

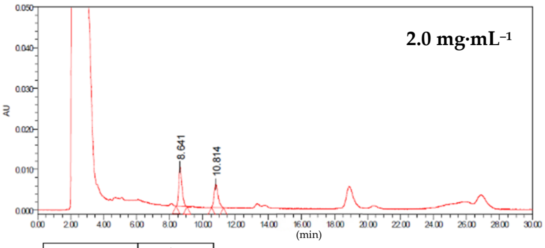

Retention time (min)	Peak area (μV*s)
8.641	129050
10.814	95129

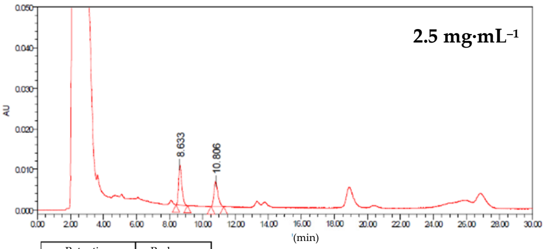

Retention time (min)	Peak area (μV*s)
8.633	133566
10.806	102747

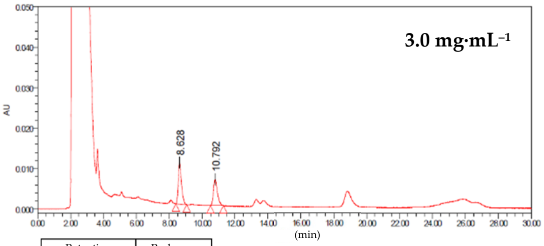

Retention time (min)	Peak area (μV*s)
8.628	137831
10.792	108727

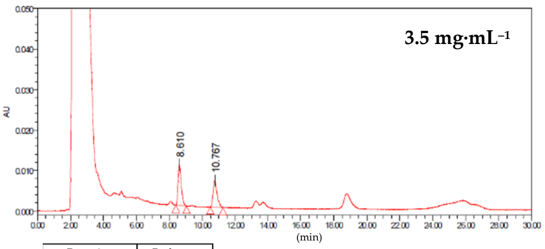

Retention time (min)	Peak area (μV*s)
8.610	136843
10.767	109409

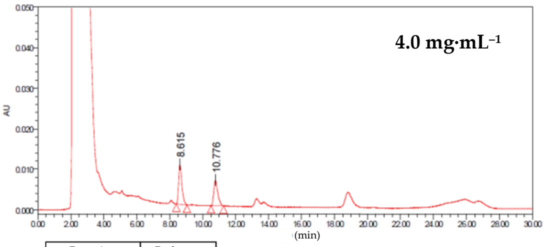

Retention time (min)	Peak area (μV*s)
8.615	133699
10.776	106792

Figure S3. HPLC chromatograms of concentrations of derivatization reagent added.

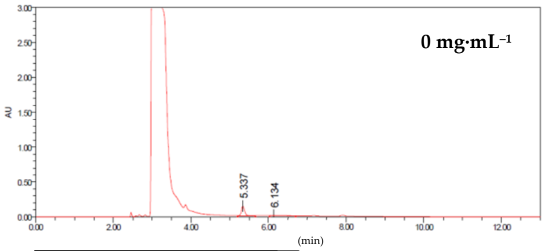

Retention time (min)	Peak area (μV*s)	Peak height (μV)
5.337	759224	137352
6.134	13483	1640

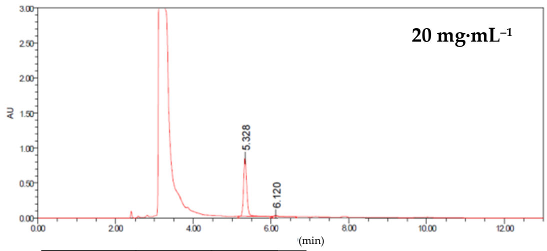

Retention time (min)	Peak area (μV*s)	Peak height (μV)
5.328	4820827	833307
6.120	111796	15537

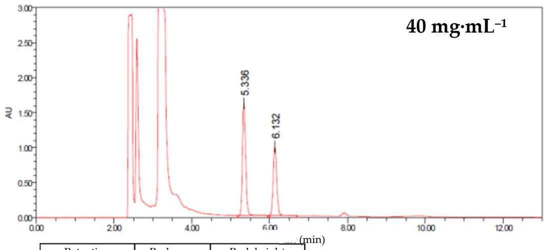

Retention time (min)	Peak area (μV*s)	Peak height (μV)
5.336	9263523	1601987
6.132	6249942	988104

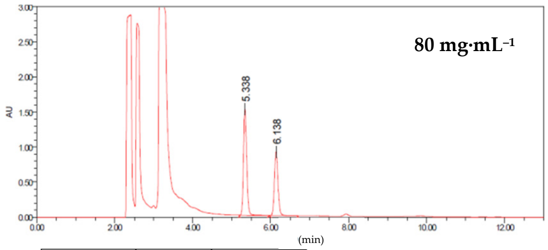

Retention time (min)	Peak area (μV*s)	Peak height (μV)
5.338	8740947	1514568
6.138	5768340	907120

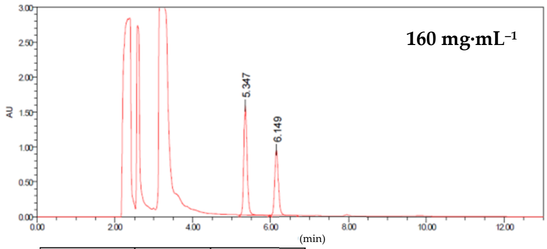

Retention time (min)	Peak area (μV*s)	Peak height (μV)
5.347	9284799	1568693
6.149	6107139	939533

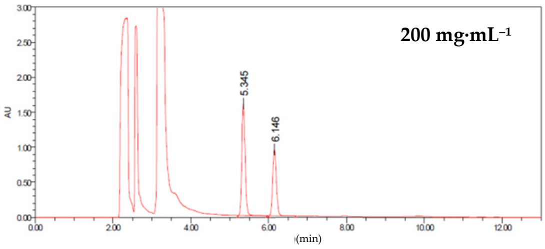

Retention time (min)	Peak area (μV*s)	Peak height (μV)
5.345	9432635	1599821
6.146	6147665	949601

Figure S4. HPLC chromatograms of concentrations of NaOH added.

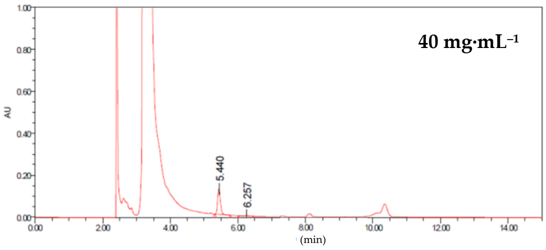

Retention time (min)	Peak area (μV*s)	Peak height (μV)
5.440	707123	122269
6.257	18463	2801

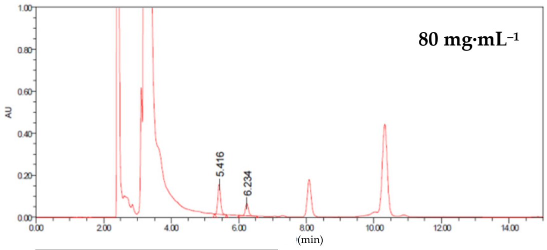

Retention time (min)	Peak area (μV*s)	Peak height (μV)
5.416	812875	141899
6.234	377760	58895

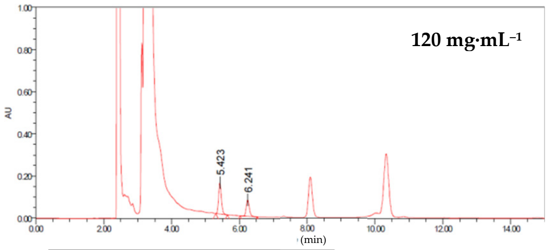

Retention time (min)	Peak area (μV*s)	Peak height (μV)
5.423	843039	148080
6.241	501225	78480

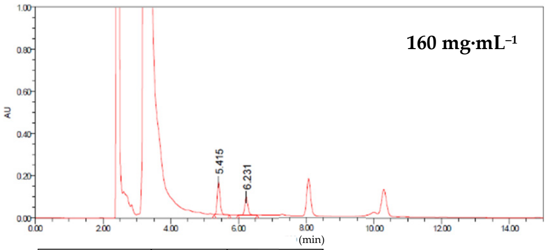

Retention time (min)	Peak area (μV*s)	Peak height (μV)
5.415	877684	154208
6.231	550517	86088

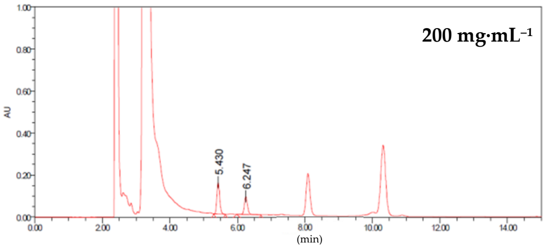

Retention time (min)	Peak area (μV*s)	Peak height (μV)
5.430	858479	150281
6.247	561525	85890

Figure S5. HPLC chromatograms of concentrations of Na_2CO_3 added.

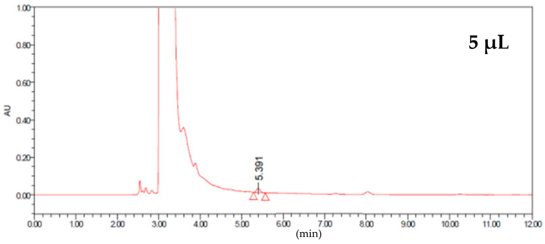

Retention time (min)	Peak area (μV*s)	Peak height (μV)
5.391	132293	22287

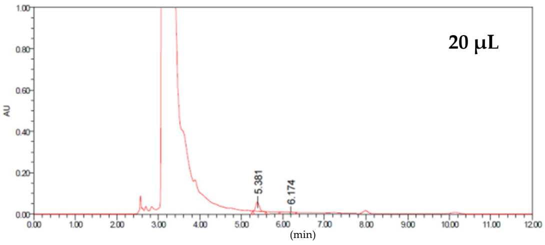

Retention time (min)	Peak area (μV*s)	Peak height (μV)
5.381	273134	46092
6.174	6319	771

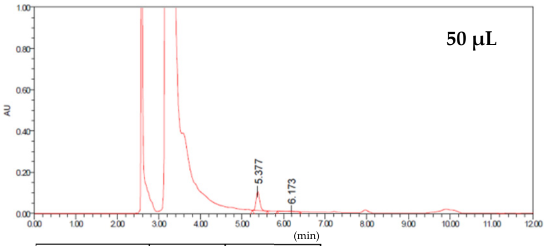

Retention time (min)	Peak area (μV*s)	Peak height (μV)
5.377	552478	91794
6.173	19842	2206

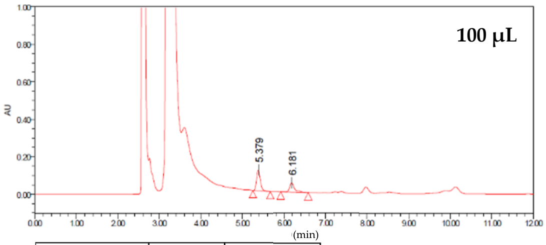

Retention time (min)	Peak area (μV*s)	Peak height (μV)
5.379	687945	109862
6.181	408197	49492

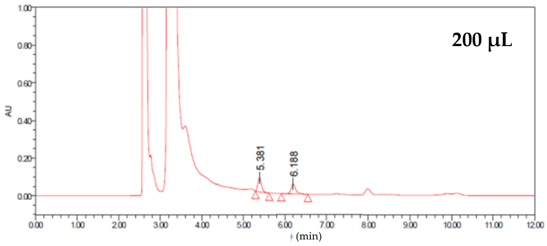

Retention time (min)	Peak area (μV*s)	Peak height (μV)
5.381	450962	73748
6.188	411125	52310

Figure S6. HPLC chromatograms of amount of Et_3N added.

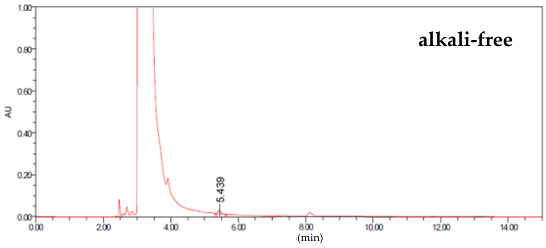

Retention time (min)	Peak area (μV*s)	Peak height (μV)
5.439	97468	17529

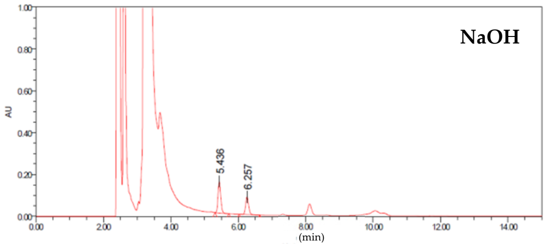

Retention time (min)	Peak area (μV*s)	Peak height (μV)
5.436	853651	150560
6.257	541104	85267

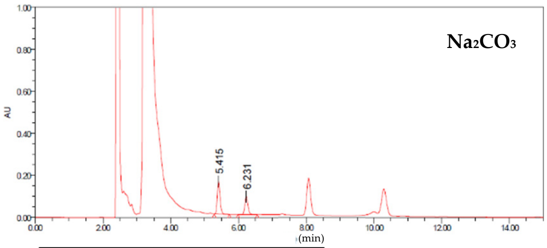

Retention time (min)	Peak area (μV*s)	Peak height (μV)
5.415	877684	154208
6.231	550517	86088

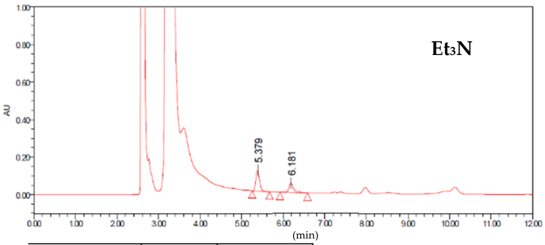

Retention time (min)	Peak area (μV*s)	Peak height (μV)
5.379	687945	109862
6.181	408197	49492

Figure S7. HPLC chromatograms of types of base.

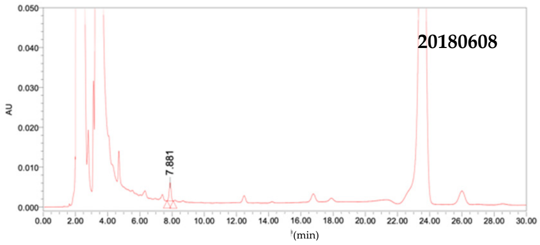

Retention time (min)	Peak area (μV*s)	Peak height (μV)
7.881	38325	4585

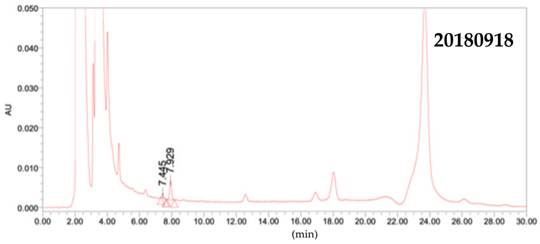

Retention time (min)	Peak area (μV*s)	Peak height (μV)
7.929	41384	4819

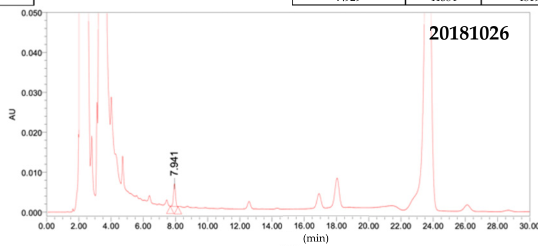

Retention time (min)	Peak area (μV*s)	Peak height (μV)
7.941	48953	5737

Figure S8. HPLC chromatograms of samples.

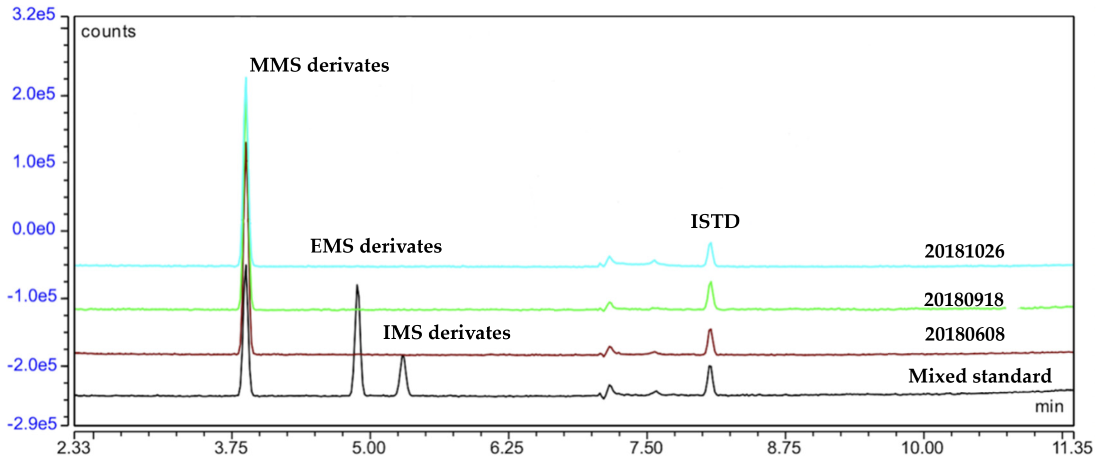

Figure S9. GC-MS chromatograms of samples
